# Acute neuromuscular, cardiovascular, and muscle oxygenation responses to low‐intensity aerobic interval exercises with blood flow restriction

**DOI:** 10.1113/EP091742

**Published:** 2024-06-14

**Authors:** Colin Lavigne, Valentin Mons, Maxime Grange, Grégory M. Blain

**Affiliations:** ^1^ Université Côte d'Azur, LAMHESS Nice France

**Keywords:** arterial blood flow, blood flow restriction, central fatigue, interval exercise, interval training, peripheral fatigue, vascular occlusion

## Abstract

We investigated the influence of short‐ and long‐interval cycling exercise with blood flow restriction (BFR) on neuromuscular fatigue, shear stress and muscle oxygenation, potent stimuli to BFR‐training adaptations. During separate sessions, eight individuals performed short‐ (24 × 60 s/30 s; SI) or long‐interval (12 × 120 s/60 s; LI) trials on a cycle ergometer, matched for total work. One leg exercised with (BFR‐leg) and the other without (CTRL‐leg) BFR. Quadriceps fatigue was quantified using pre‐ to post‐interval changes in maximal voluntary contraction (MVC), potentiated twitch force (QT) and voluntary activation (VA). Shear rate was measured by Doppler ultrasound at cuff release post‐intervals. Vastus lateralis tissue oxygenation was measured by near‐infrared spectroscopy during exercise. Following the initial interval, significant (*P *< 0.05) declines in MVC and QT were found in both SI and LI, which were more pronounced in the BFR‐leg, and accounted for approximately two‐thirds of the total reduction at exercise termination. In the BFR‐leg, reductions in MVC (–28 ± 15%), QT (–42 ± 17%), and VA (–15 ± 17%) were maximal at exercise termination and persisted up to 8 min post‐exercise. Exercise‐induced muscle deoxygenation was greater (*P *< 0.001) in the BFR‐leg than CTRL‐leg and perceived pain was more in LI than SI (*P *< 0.014). Cuff release triggered a significant (*P *< 0.001) shear rate increase which was consistent across trials. Exercise‐induced neuromuscular fatigue in the BFR‐leg exceeded that in the CTRL‐leg and was predominantly of peripheral origin. BFR also resulted in diminished muscle oxygenation and elevated shear stress. Finally, short‐interval trials resulted in comparable neuromuscular and haemodynamic responses with reduced perceived pain compared to long‐intervals.

## Abstract (highlights)

- **What is the central question of this study?**
  Does modifying exercise–rest durations during low‐intensity blood‐flow‐restricted (BFR) interval aerobic exercise modulate neuromuscular fatigue development, shear stress and muscle oxygenation?
- **What is the main finding and its importance?**
  BFR interval exercise resulted in significantly greater neuromuscular fatigue, predominantly of peripheral origin, compared to exercise with unrestricted blood flow. Furthermore, shorter BFR intervals elicited comparable neuromuscular and haemodynamic responses compared to longer intervals. These findings have important implications for developing optimized BFR training interventions and for future studies assessing whether these acute stimuli translate into enhanced physiological adaptations and subsequent improvements in performance.

## 1 INTRODUCTION

Exercise combined with blood flow restriction (BFR) has been used for several decades (Sato, 2005) with the main purpose of improving muscular hypertrophy and strength (Lixandrão et al., 2018; Takarada et al., 2002; Vechin et al., 2015). Recently, BFR as an adjunct to aerobic exercise has emerged as an effective training paradigm to enhance improvements in skeletal muscle and cardiovascular adaptations (Christiansen, Eibye, Rasmussen et al., 2019, Christiansen et al., 2020). This modality typically uses a pneumatic cuff to partially restrict arterial blood flow and obstruct venous outflow of the exercising limb(s) (Patterson et al., 2019). BFR applied intermittently (i.e., regular cuff inflations/deflations during exercise work/rest periods, respectively) results in local hypoxaemia and exacerbated intramuscular metabolic perturbations (Horiuchi & Okita, 2012; Manini & Clark, 2009), whereas cuff deflations result in local reactive hyperaemia (Gundermann et al., 2012) facilitating reperfusion and thus elevating endothelial shear stress (Christiansen, Eibye, Rasmussen et al., 2019). Together, the transient periods of cellular homeostatic disruptions and fluctuations in shear stress patterns to cuff inflations and deflations create a powerful framework to augment mitochondrial biogenesis (Keramidas et al., 2012) and angiogenesis (Ferguson et al., 2018; Mitchell et al., 2019), which collectively might contribute to improved endurance exercise performance (Ferguson et al., 2021). A few other favourable mechanisms underlying the adaptations to BFR exercise include reactive oxygen species production (Christiansen, Eibye, Hostrup et al., 2019), increased type 2 muscle fibre recruitment (Loenneke & Pujol, 2009) and greater activation of molecular signalling processes (Ferguson et al., 2021; Loenneke et al., 2011). Conditions involving low blood flow (as seen with BFR) produce high metabolic stress (e.g., accumulation of inorganic phosphate, hydrogen ions, potassium, etc.) (Loenneke et al., 2011; Pearson & Hussain, 2015; Suga et al., 2010), thereby reducing exercise tolerance and accelerating neuromuscular fatigue compared to work‐matched training without BFR (Broxterman et al., 2015).

Classically, neuromuscular fatigue can be defined as a transient exercise‐induced reduction in the ability of a muscle to produce force or power (Bigland‐Ritchie & Woods, 1984). In this context, exercise‐induced neuromuscular fatigue involves peripheral (i.e., biochemical changes within the active muscle) (Allen et al., 2008) and central mechanisms (i.e., failure of the central nervous system to voluntarily activate muscle) (Gandevia, 2001) that can be quantified non‐invasively with electrical stimulation of the motor nerve (Merton, 1954). In previous research investigating changes in neuromuscular fatigue following aerobic exercise with superimposed BFR at 80% of limb occlusion pressure, maximal voluntary torque was ∼35% lower in the BFR condition compared to work‐matched exercise without BFR 1 min after exercise (Kilgas et al., 2022). These reductions in maximal voluntary torque appeared to be influenced by peripheral mechanisms, as indicated by the pronounced exercise‐induced reduction in evoked twitch force with BFR. However, this work was limited to pre‐ and post‐exercise neuromuscular measurements and little is known about fatigue development during exercise (Kilgas et al., 2022). Moreover, studies reporting on the physiological responses to aerobic interval exercise with BFR have been limited to protocols involving 2 min of exercise separated by 60 s of recovery (Christiansen et al., 2018; Corvino et al., 2017; Kilgas et al., 2022). It is, however, possible that protocols with multiple shorter work intervals (i.e., 1 min) and recovery (i.e., 30 s) may mitigate metabolic perturbations (i.e., less neuromuscular fatigue) compared to work‐matched longer intervals (i.e., 2 min) and recovery (i.e., 60 s) (Fiorenza et al., 2019; McClean et al., 2023) and effectively decrease the potential discomfort associated with BFR and, therefore, augment exercise tolerance during BFR training. Conversely, diminishing the duration of exposure to localized ischaemia may lead to a decrease in hypoxic and metabolic stresses during exercise, as well as shear stress at cuff release, potentially weakening the angiogenic and mitochondrial adaptations that typically follow BFR training. The effect of interval exercise/rest durations on neuromuscular fatigue, muscle oxygenation, shear rate and perceived pain remains yet to be determined.

Indeed, past research has primarily concentrated on optimizing BFR protocols by manipulating exercise intensity (Suga et al., 2010) and restrictive pressure (Fatela et al., 2016; Kilgas et al., 2022; Reis et al., 2019; Singer et al., 2020). Findings indicated that, during exercise with BFR, increases in exercise intensity and/or restrictive pressure exhibit a dose–response relationship with the metabolic and cardiovascular stressors that occur in response to exercise. For example, increasing restrictive pressures to exercising muscles results in decreased blood flow during BFR‐exercise (Singer et al., 2020), increased tissue deoxygenation (Reis et al., 2019; Singer et al., 2020), and greater neuromuscular impairments (Kilgas et al., 2022). However, it is unclear whether this dose–response pattern translates to similar cardiovascular haemodynamic, muscle oxygenation and neuromuscular changes by manipulating interval durations and/or the recovery periods characterizing intermittent exercise. Determining optimal interval durations regarding BFR exercise might help elucidate the physiological responses to this modality and provide valuable insight for maximizing training prescription and outcomes following BFR training.

The purpose of this study was to examine the development of neuromuscular fatigue and provide insight from a cardiovascular haemodynamic and muscle oxygenation perspective during and after short‐interval (SI) and long‐interval (LI) aerobic BFR exercise. For these experiments, we used unilateral leg BFR during SI and LI exercise on a cycling ergometer. From a neuromuscular perspective, we simultaneously performed identical neuromuscular measurements in the contralateral control limb of each subject, who exercised in a free‐flow condition, to further investigate the effects of exercise with BFR to the same exercise without BFR. We hypothesized that (1) after intervals at time‐matched periods, exercise with BFR would accelerate indices of neuromuscular fatigue compared to exercise without BFR; and (2) due to a possible dose–response relationship, the protocol including multiple longer intervals would induce increases in deoxygenated haemoglobin concentration during exercise and an exaggerated increase in arterial blood flow and shear stress at cuff release.

## 2 METHODS

### 2.1 Ethical approval

This study was approved by the Committee for the Protection of Persons West III (reference number: 2022‐A01627‐36) and was conducted in accordance with the standards set by the latest revision of the *Declaration of Helsinki*, except for registration in a database. All participants provided written informed consent.

### 2.2 Participants

Eight participants completed the study (see characteristics in Table 1). Participants were: (1) free from diagnosed cardiovascular, musculoskeletal or metabolic diseases; (2) normotensive; (3) self‐identified non‐smokers; (4) not taking any medications; and (5) involved in regular exercise (range: recreational activity to competitive amateur sport). The present study was powered to address the first research hypothesis that BFR exercise would accentuate neuromuscular fatigue. Using a large effect size (partial η^2^ = 0.14) due to the large effect BFR exercise has on muscle fatigue development (Husmann et al., 2018), an a priori power analysis performed on the G*power software program (version 3.1) revealed that at least six participants would be required (α = 0.05, β = 0.8, non‐sphericity correction = 1, ANOVA with repeated‐measures within–between interaction).

**TABLE 1:** Participant characteristics and peak physiological parameters measured during the step‐incremental test on the cycle‐ergometer.

Outcome measure	Data (*n* = 8)
Characteristics	
Age (years)	23 ± 2
Male, *n* (%)	6 (75)
Female, *n* (%)	2 (25)
Height (m)	1.76 ± 0.09
Weight (kg)	71.4 ± 17.0
Body mass index (kg m^−2^)	22.7 ± 8.3
SBP (mmHg)	125 ± 16
DBP (mmHg)	65 ± 5
VL skinfold (mm)	14.6 ± 8.7
Step‐incremental exercise	
*W*_max_ (W)	268 ± 73
${\dot{V}}_{O_{2}\max}$ (L min^–1^)	3.30 ± 0.83
${\dot{V}}_{O_{2}\max}$ (mL kg^–1^ min^–1^)	46.5 ± 5.3
${\dot{V}}_{\mathrm{Emax}}$ (L min^–1^)	123 ± 32
RER	1.12 ± 0.07
HR_max_ (beats min^–1^)	186 ± 6
Peak blood lactate (mmol L^–1^)	12.3 ± 3.5

*Note*: Values are expressed as means ± SD. Abbreviations: DBP, diastolic blood pressure; HR_max_, maximum heart rate; RER, respiratory exchange ratio; SBP, systolic blood pressure; ${\dot{V}}_{E},$ minute ventilation; VL, vastus lateralis; $\dot{V}o_{2\max},$ maximal rate of O_2_ consumption; *W*_max_, peak power output.

### 2.3 Study design

Participants visited the laboratory on six separate occasions to perform: (1) a one step‐incremental cycling test to exhaustion for determination of maximal O_2_ consumption (${\dot{V}}_{O_{2}\max}$) and maximal power output (*W*_max_); (2) one familiarization session to become familiar with BFR exercise, neuromuscular testing procedures and perceptual ratings; and (3) two interval exercise regimes performed twice in random order over four experimental visits. The legs of participants were randomly assigned to cycling either without (CTRL‐leg) or with (BFR‐leg) blood flow restriction. All exercise was performed on an electromagnetically braked cycle ergometer (Velotron, Elite Model; Racer Mate Inc., Seattle, WA, USA) equipped with power meter pedals (Assioma Duo, Favero Electronics, Arcade, Italy). During each session, the power generated by each leg (range: 45%–55%) and cadence (maintained at 85 ± 5 rpm) was displayed on a handlebar‐mounted bike computer (Garmin Edge 810, Garmin Ltd, Olathe, KS, USA). Task failure criteria consisted of failing to maintain equal power output between legs (i.e., a differential >10% for ≥10 s) or cadence (i.e., a drop ≤10 rpm for ≥10 s) within the target range despite strong verbal encouragement. All sessions were performed at the same time of day (±1 h) in an environmentally controlled room (temperature: ∼20°C; relative humidity 40%–50%) and separated by ≥72 h. Prior to each test, participants were asked to refrain from strenuous physical activity for 24 h and avoid consumption of food and caffeine for 2 and 12 h, respectively.

### 2.4 Step‐incremental cycling test

On the first visit, participant characteristics including, age (years), height (cm), and body mass (kg) were recorded (see Table 1). Subsequently, participants completed a step‐incremental test. The step‐incremental protocol was initiated with an external workload of 50 or 70 W, followed by an incremental phase of 15 or 20 W min^–1^ depending on the physical ability of the participant. Participants were instructed to maintain a pedal rate of 80–90 rpm. Exercise was terminated when participants reached volitional exhaustion or could no longer maintain 60 rpm for more than 10 s despite verbal encouragement. To ensure that participants reached ${\dot{V}}_{O_{2}\max}$ during the step‐incremental protocol, participants performed, after a 20‐min recovery period, a constant workload test until exhaustion at 110% of *W*_max_. In all participants, the peak ${\dot{V}}_{O_{2}}$ from the constant workload trial was different (*P* = 0.037) from the peak ${\dot{V}}_{O_{2}}$ from the maximal and graded exercise test (44.7 ± 6.1 vs. 46.5 ± 5.3 mL min^−1^ kg^−1^, respectively), indicating that not every participant reached ${\dot{V}}_{O_{2}\max}$ during the maximal and graded exercise test. The ergometer position was recorded and subsequently replicated.

### 2.5 Familiarization trial

Arterial occlusion pressure (AOP), defined as the point when blood velocity was no longer detected using Doppler ultrasound, was measured once in triplicate in each participant during the familiarization visit, and kept consistent over the entire duration of the experiment. AOP was determined seated at rest, with 2 min of rest between trials, by progressively inflating a 13 × 85 cm pneumatic cuff (Hokanson Inc., Bellevue, WA, USA) placed around the most proximal region of the thigh, connected to an air source and rapid inflation system (E20 AG101, Hokanson). After AOP assessment, participants practiced multiple 1‐ and 2‐min sets of low‐intensity (i.e., 40% *W*_max_) whole‐body cycling exercise with (BFR‐leg) or without (CTRL‐leg) BFR set to 80% AOP. Participants were also thoroughly familiarized with the neuromuscular testing procedures and perceptual measures.

### 2.6 Experimental trials

The experimental trials included two exercise protocols matched for the exercise‐to‐recovery ratio (2:1) and total work (24 min), but different in interval and recovery duration. SI included 24 × 60 s exercise intervals interspersed with 30 s of passive recovery, whereas LI included 12 × 120 s exercise intervals interspersed with 60 s of passive recovery. The intensity of intervals were 40% *W*_max_ (107 ± 28 W, 85 rpm) and blood flow was restricted by applying 80% AOP (pressure: 171 ± 31 mmHg) to the BFR‐leg. This percentage was chosen because no significant change in limb tissue deoxygenation is observed at higher percentage AOP (Singer et al., 2020). The pneumatic cuff was placed around the proximal lower limb and rapidly inflated and completely deflated at the start and end of each work interval, respectively. Interval protocols were preceded by a standardized warm‐up consisting of 5 min of continuous cycling without BFR at a workload corresponding to 50% of the *W*_max_ (122 ± 59 W), followed by 5 min of rest.

Neuromuscular function assessments were performed at baseline, after the first interval, and after every 2 min of work (i.e., two and one intervals in SI and LI, respectively) during exercise, and right after the end of exercise. To capture the rapid recovery from fatigue that occurs within seconds after exercise cessation, all participants performed a neuromuscular function assessment protocol (see ‘Neuromuscular variables’ below) precisely at the 10‐s mark following exercise cessation. To ensure all participants could efficiently and quickly transition between the cycle ergometer and the isometric dynamometer with investigator assistance, several measures were implemented. The cycle ergometer was positioned ∼1 m perpendicular from the isometric dynamometer. First, all equipment essential for BFR and neuromuscular function testing remained affixed to the participants throughout the entire duration of the experiment. Wires and pneumatic tubing, each measuring over 3 m in length, were strategically positioned to hang from the ceiling, ensuring no interference during transitions between cycling and isometric dynamometer exercises. Second, non‐compliant straps, each fitted with a metal ring, were securely fastened to both the right and left ankles of participants. During transitions from the cycle ergometer to the isometric dynamometer, this ring was readily connected and disconnected to a force transducer equipped with a hook. Finally, the participants' hips and torso were not secured to the ergometer with straps. Instead, participants were instructed to maintain contact between their glutes and shoulders with the ergometer, while placing their hands at hip height and gripping handles on the same side to minimize extraneous movements of the upper body. Surface electromyography (EMG) data were obtained continuously from vastus lateralis (VL), vastus medialis (VM) and rectus femoris (RF) muscles. Pulmonary gas exchange and muscle tissue oxygenation were measured continuously.

A second set of experimental trials was performed to characterize the impact of BFR exercise on central haemodynamics and local haemodynamics during SI and LI. First, participants rested for a minimum of 10 min to facilitate baseline cardiovascular assessments. Arterial blood flow measurements in the BFR‐leg were performed at baseline, immediately after every 2 min interval, and immediately after the end of the protocol to 8 min (i.e., following the same pattern as neuromuscular assessments). Heart rate, systolic, diastolic and mean arterial pressure (MAP) were simultaneously recorded.

### 2.7 Data collection and analyses

#### 2.7.1 Neuromuscular variables

Participants were placed in a custom‐built isometric chair with both trunk–thighs and knee joints at angles of 90°. Non‐compliant straps attached to calibrated load cells (LC101‐500 Omegadyne, Sunbury, OH, USA) were connected to the participants’ right and left ankles, just superior to both ankle malleoli. Neuromuscular procedures began with determination of femoral nerve stimulation intensity. Peripheral stimuli to the femoral nerves were delivered via surface cathodes (3 cm × 3 cm, Ag/AgCl, Mini‐KR, Contrôle‐Graphique, Brie‐Comte‐Robert, France) and rectangular anodes (50 × 90 mm, Durastick Plus; DJO Global, Vista, CA, USA) placed onto the skin over the femoral triangles and gluteal folds, respectively. A constant‐current stimulator (DS7A, Digitimer, Welwyn Garden City, UK) delivered a square‐wave stimulus (1 ms) at a maximum of 400 V. Electrical stimuli were administered at rest in 10 mA stepwise increments from 10 mA until maximal quadriceps twitch force and compound muscle action potential (*M*_max_) for the VL, VM and RF of each limb were achieved. The resulting stimulation intensity was further increased by 30% (average supramaximal intensity: 76 ± 18 mA) to ensure full spatial motor unit recruitment during the neuromuscular procedures (Neyroud et al., 2014). The positions of surface cathodes and anodes were marked with indelible ink to ensure reproducible stimulation sites across visits. Surface cathodes were then securely tapped to the skin to ensure they remained in place throughout each experimental session. Following a standardized warm‐up of five 3 s submaximal isometric contractions at 10%, 25%, 50%, 75% and 90% of estimated maximal voluntary contraction (MVC), separated by rest periods of 30 s, baseline quadriceps neuromuscular function was assessed. In each neuromuscular assessment, participants performed a 3 s MVC during which superimposed paired stimuli at 100 Hz (QT_100,superimposed_) were delivered at the peak force of the MVC to determine voluntary activation (VA). Then, potentiated quadriceps twitch forces evoked by paired 100 Hz (QT_100_), paired 10 Hz (QT_10_) and single (QT_single_) electrical stimulations of the femoral nerve were elicited 2, 4 and 6 s after each MVC, respectively. At baseline (i.e., before the cycling trials), two MVCs without stimulation preceded the neuromuscular assessments. All MVCs were separated by 1 min. During MVCs, participants were instructed to place their hands at hip height, so that they were holding onto handles ipsilaterally to avoid extraneous movements of the upper body. During the exercise trials, measurements were recorded from a single neuromuscular evaluation beginning at 10 s into the rest periods. Postexercise measurements were then recorded 10 s, 1, 2, 4 and 8 min after the exercise trials. Data were recorded at 1 kHz in LabChart software (v8.1.25, ADInstruments, Bella Vista, Australia).

Neuromuscular fatigue was determined as the exercise‐induced decline in MVC force (global fatigue), in QT_100_, QT_10_ and QT_single_ (peripheral fatigue) and in VA (central fatigue). MVC force was determined as the highest voluntary force achieved during the baseline MVCs. The amplitude of QT_100_, QT_10_ and QT_single_ was measured, and VA was calculated according to the formula: $\mathrm{VA}\;\left(\%\right)=\;\left(1-\mathrm{superimposed}\;QT_{100}/QT_{100}\right)\times 100$ (Merton, 1954). Maximal rate of force development (MRFD; maximal value of the first derivative of the force single) and half‐relaxation time (HRT; time to obtain half the decline in maximal force) were assessed for QT_single_. The ratio between QT_10_ and QT_100_ (QT_10:100_) was calculated as an index of prolonged low‐frequency force depression (Place et al., 2010). Post‐exercise values were normalized as a percentage of pre‐exercise values.

#### 2.7.2 Surface electromyography variables

Electromyographic (EMG) signals were detected using wireless EMG sensors (Trigno EMG, Delsys Inc., Boston, MA, USA) with an inter‐electrode spacing of 10 mm. Sensors were positioned parallel to the muscle fibres over the centre of the muscle bellies of the VL, VM, RF, and long head of biceps femoris (BF). The area of skin under each electrode was shaved, lightly abraded and cleaned with an isopropyl alcohol swab to reduce skin impedance below 3 kΩ. Tape was used to keep the EMG sensors in place. Attention as to the positions of these electrodes was given to ensure *M*_max_ amplitudes were optimized, and the position of these electrodes were marked with indelible ink to ensure reproducible measures across visits.

The EMG sensors have a common‐mode rejection ratio of 85 dB and band‐pass filter of 20–450 Hz. EMG signals were sampled at 2000 Hz utilizing LabChart software (version 8, ADInstruments). Electrically evoked *M*_max_ responses of VL, VM and RF muscles were analysed for peak‐to‐peak amplitude, defined as the absolute difference from the lowest deflection to the highest inflection of the biphasic wave. The root mean square of the EMG signal recorded during each MVC (RMS_MVC_) was analysed as the highest 500 ms average produced during voluntary effort. *M*_max_ and RMS parameters were calculated to evaluate changes in peripheral neuromuscular excitability and changes in voluntary muscle activation, respectively. During exercise, the RMS of each burst from the VL, VM and RF was analysed using a custom‐written script in MATLAB (The MathWorks, Natick, MA, USA), as previously reported (Ducrocq et al., 2021). Briefly, the algorithm filtered, rectified and smoothed the EMG signal and a minimum threshold was applied to determine the onset and offset of the bursts. The RMS of each burst from the EMG signal was then calculated, normalized to the maximum RMS_MVC_ recorded during the baseline neuromuscular assessments. For data analysis, each burst was averaged into windows of 25%, 50%, 75% and 100% of each interval. Aberrant data that were ±3 SD outside the local mean were removed.

#### 2.7.3 Blood flow and haemodynamic variables

Blood flow was measured in the superficial femoral artery of the BFR‐leg only, just distal to the pneumatic pressure cuff. Blood velocity and vessel diameter were measured with a duplex ultrasound imaging device (ArtUs EXT‐1H, Telemed Medical Systems, Vilnius, Lithuania). The ultrasound system was equipped with a linear array transducer (L12‐5N40‐A4, Telemed Medical Systems) and operating at a B‐mode imaging frequency of 12 MHz and Doppler velocity frequency of 5 MHz using EchoWave II software (version 3.4.4, Telemed Medical Systems). Blood velocity measurements were obtained with the probe positioned to maintain an insonation angle of ≤60° to the axis of the vessel and the sample volume (range: 1–5 mm) cantered and maximized according to artery size. Probe position of the ultrasound was marked with indelible ink to ensure measurement accuracy and repeatability. Three ultrasound recordings were obtained at rest which were averaged for analysis. During these protocols, blood flow was recorded after every 2 min of work (i.e., every two and one intervals for SI and LI, respectively) by having participants transition their foot from the ergometer pedal to a stool while the pneumatic cuff remained inflated for this short period (range: 4–8 s). Once the probe was over top of the artery, recording began, and the cuff was rapidly deflated to 0 mmHg. Doppler images of blood flow were also obtained immediately, 1, 2, 4 and 8 min after exercise. Systolic (SBP), diastolic (DBP) and mean arterial (MAP) blood pressure were measured in parallel with velocity using finger photoplethysmography (Finapres Medical Systems, Amsterdam, The Netherlands) with inflatable cuffs on the index and middle finger of the left hand and recorded to Labchart software (v.8.1.25, ADInstruments).

Mean blood velocity was identified from the pulse wave spectrum recording as the greatest three cardiac cycles following cuff release. Vessel diameter was obtained by measuring the perpendicular distance between the superficial and deep walls on the B‐mode image. Blood flow was calculated as: $\mathrm{blood}\;\mathrm{flow}\;=\;\mathrm{mean}\;\mathrm{blood}\;\mathrm{velocity}\;\times \;\pi \;\times \;{\left(\frac{\mathrm{vessel}\;\mathrm{diameter}}{2}\right)}^{2}\;\times \;60\;$(Wray et al., 2011). MAP was calculated as 1/3 (SBP – DBP) + DBP. Shear rate was calculated as four times velocity divided by diameter (Pyke et al., 2004). Vascular conductance was calculated as the quotient of simultaneously measured blood flow and MAP (Limberg et al., 2020).

#### 2.7.4 Near‐infrared spectroscopy variables

Participants were instrumented with near‐infrared spectroscopy (NIRS) probes (Oxymon, Artinis Medical Systems, Elst, The Netherlands), using a two‐wavelength continuous system (i.e., 761 and 845 nm) to obtain oxygenated, deoxygenated and total haemoglobin and a differential path‐length factor of 4.0 (Van Beekvelt et al., 2002). The NIRS probes were fixed longitudinally over the VL muscle bellies, approximately two‐thirds between the greater trochanter and superior border of the patella, proximal to the surface EMG sensors (Lucero et al., 2018). The probes were connected by fibre‐optic cables to 90° optodes at the tip and consisted of source detector separations between 2.5 and 5.5 cm. Probes were secured in place by tape and a tensor bandage to limit any extraneous light or movement from interfering with the device. Data were recorded at a sample rate of 50 Hz using OxySoft version 2.1.6 software (Artinis). The area of the skin underneath the NIRS probe was prepared by shaving excess hair and cleaning with an isopropyl alcohol swab. The site of the NIRS probe was marked to ensure consistent placement between sessions. Skinfold thickness was measured at the area of the NIRS sensor placement using a Harpenden skin fold caliper to account for skin and subcutaneous adipose tissue thickness. The optodes were adjusted to measure more than double the distance of the skinfold thickness (14.6 ± 8.7 mm) underneath the probe (Barstow, 2019). The data were extracted from the NIRS software (OxySoft 2.1.6, Artinis) and a customized script (MATLAB) analysed the data. Oxyhaemoglobin, deoxyhaemoglobin and total haemoglobin concentrations were averaged using a 3 s time window and normalized to reflect changes from the resting baseline. Deoxyhaemoglobin provides a reliable measure of changes in muscle deoxygenation status due to O_2_ extraction (Hoshi et al., 2001), thus offering an estimate of changes in intramuscular oxygenation status (Ferrari et al., 1997). The change in concentrations during each interval were assessed by calculating the difference between the maximum value during each exercise period and the last 3 s of data recorded prior to beginning the next exercise bout and inflating the cuff. The deoxygenated NIRS signal was used to determine the time spent at an intensity greater than 90% of the maximum value observed for a given participant.

#### 2.7.5 Metabolic and respiratory variables

Oxygen uptake (${\dot{V}}_{O_{2}}$), minute ventilation (${\dot{V}}_{E}$), the ventilatory equivalent for carbon dioxide (${\dot{V}}_{E}$ /), respiratory exchange ratio (RER), breathing frequency (*f*_B_) and tidal volume (*V*_T_) were measured breath‐by‐breath using a portable gas analyser (Cortex MetaMax 3B, Cortex GmbH, Leipzig, Germany). A calibration of the gas analysers and manual volume calibration of the pneumotachograph with a 3‐L syringe were performed prior to each protocol. Data were recorded and stored with MetaSoft Studio (Cortex). Participants breathed through a silicone rubber facemask (7450 series, Hans Rudolph Inc., Shawnee, KS, USA). Heart rate (HR) was collected using a HR monitor (Polar H7, Polar Electro Oy, Kempele, Finland).

All peak ventilatory variables were computed from the highest 10‐s rolling average during each interval. For the ramp‐incremental test, peak ventilatory variables were determined from the highest 30‐second rolling average. For the constant workload validation test, they were determined from the highest 10‐second rolling average. Analysis of the validation test was used to confirm attainment of a true ${\dot{V}}_{O_{2}\max}$, which used the criterion that the ${\dot{V}}_{O_{2}}$ value from both tests did not differ by >3% (Poole & Jones, 2017). Blood lactate (mmol L^−1^) concentrations were obtained from a 5 μL sample of blood taken from a finger‐prick at 3 min post‐exercise. Samples were analysed immediately via a portable laboratory lactate device (LactatePro; Arkray Inc., Jap).

#### 2.7.6 Perceptual variables

Rate of perceived exertion (RPE) was determined using the Borg CR 6–20 scale (Borg, 1985) and was explained according to published instructions (Borg, 1998), with 6 being no exertion at all and 20 being maximal exertion. Local muscle pain experienced in the BFR‐leg was quantified using a visual analogue pain scale (McCormack et al., 1988), with 0 being no pain at all and 10 being worst possible pain. Participants reported perceptual measurements every minute during exercise.

### 2.8 Statistical analysis

All data were tested for normal distribution using the Shapiro–Wilk test. The Greenhouse–Geisser correction factor was applied for sphericity violations. The main statistical model for this study was a three‐way ANOVA with repeated measures comparing neuromuscular function between trial (SI vs. LI), leg (CTRL‐leg vs. BFR‐leg) and time. Accordingly, separate three‐way 2 × 2 × 13 (trial × leg × time) repeated measures ANOVAs were conducted to assess differences in neuromuscular function data during exercise. Three‐way 2 × 2 × 5 (trial × leg × time) repeated measures ANOVAs assessed recovery kinetics after exercise in all neuromuscular function data. Additionally, three‐way ANOVAs with repeated measures were conducted to evaluate the effect of trial, leg and time on changes in oxygenated haemoglobin, deoxygenated haemoglobin, total haemoglobin, and RMS for each muscle (VL, VM and RF). Two‐way 2 × 2 (trial (SI vs. LI) × leg (CTRL‐leg vs. BFR‐leg)) repeated measures ANOVAs were conducted to assess differences in all normalized neuromuscular function data after the first interval during exercise. Two‐way time × trial ANOVAs with repeated measures were conducted for cardiovascular and cardiorespiratory variables. Effect sizes for each ANOVA are presented as partial eta squared (η_p_^2^), with <0.06, 0.06–0.14 and >0.14 indicating a small, medium and large effect, respectively (Cohen, 1988). If significant main effects were found, subsequent *post‐hoc* analysis was performed using the Tukey's HSD test. For *post hoc* comparisons, effect size was based on Cohen's *d*_av_, and interpretation of the effect size was considered as small (0.2 ≤ *d* < 0.5), medium (0.5 ≤ *d* < 0.8) or large (*d* ≥ 0.8) (Cohen, 1988). Statistical analyses were performed using Jamovi statistical software (jamovi, version 2.5, 2024; retrieved from https://www.jamovi.org). Illustrations were created using GraphPad Prism (v.10; GraphPad Software, Boston, MA, USA). Statistical significance was accepted at *P* < 0.05. All data are presented as means ± standard deviation (SD).

## 3 RESULTS

### 3.1 Quadriceps muscle EMG and work during exercise

There were no statistically significant three‐way interactions, two‐way interactions (trial × time, leg × time, and trial × leg), or main effects for trial, leg and time observed in either the VL, VM or RF muscle EMG during exercise. Despite instructions, familiarization and real‐time feedback, the contribution of power output was more in the CTRL‐leg than the BFR‐leg during both LI (55 ± 3.8% vs. 45 ± 3.8%, *P* = 0.004, *d* = 2.63) and SI (55 ± 2.0% vs. 45 ± 2.0%, *P* < 0.001, *d* = 5.0).

### 3.2 Neuromuscular responses to exercise protocols

**FIGURE 1:**
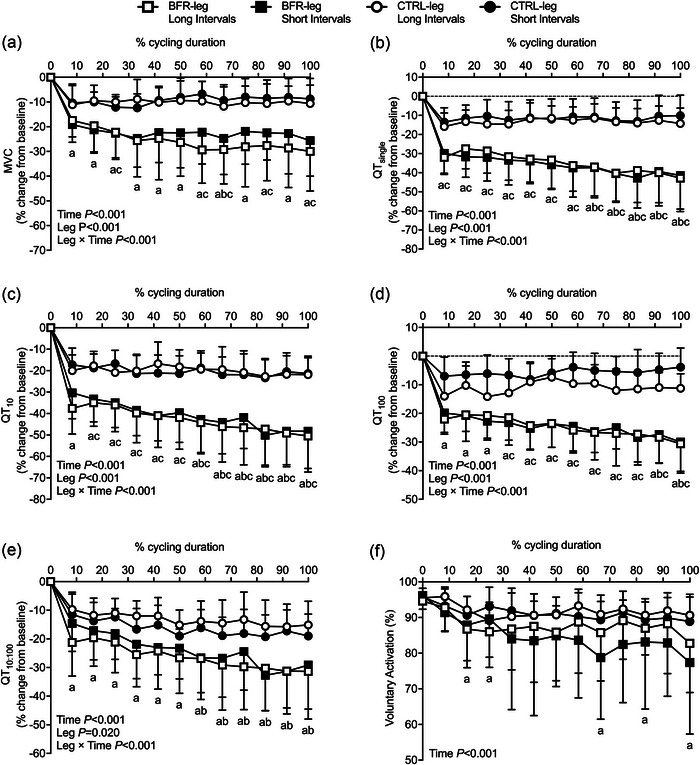
Effect of short‐interval and long‐interval trials, with (BFR‐leg) or without (CTRL‐leg) blood flow restriction throughout exercise, on maximal voluntary contraction (MVC, a), on potentiated twitch peak force evoked by single (QT_single_, b), 10 Hz (QT_10_, c) and 100 Hz (QT_100_, d) paired electrical stimulation, on prolonged low‐frequency force depression index (QT_10:100_, e) and on voluntary activation (f). Statistically significant main effects and interaction effects are indicated on the graphs (*n* = 8). *Note*: there was no main effect of trial (i.e., short‐interval vs. long‐interval) in any of these variables. ^a^ *P* < 0.05 versus baseline in BFR‐legs and CTRL‐legs; ^b^ *P* < 0.05 versus the first quarter of the protocol (i.e., first‐, second‐ and/or third time‐matched neuromuscular assessment) in BFR‐legs; ^c^ *P* < 0.05 for BFR‐legs compared with CTRL‐legs.

No differences were found for any measure at baseline between conditions (Table 2). No statistically significant three‐way interactions, two‐way trial × leg interactions or main effect of trial were observed for any neuromuscular fatigue variables. BFR‐leg SI and BFR‐leg LI displayed greater overall reductions in MVC (*P* = 0.046, *d* = 1.54; *P* = 0.016, *d* = 1.45), QT_single_ (*P* = 0.005, *d* = 2.23; *P* < 0.001, *d* = 2.04), QT_10_ (*P* = 0.003, *d* = 2.19; *P* = 0.009, *d* = 1.98), QT_100_ (*P* = 0.003, *d* = 2.51; *P* < 0.001, *d* = 2.89) and MRFD (*P* = 0.002, *d* = 2.15; *P* = 0.008, *d* = 1.94) throughout the course of exercise compared with CTRL‐leg SI and CTRL‐leg LI, respectively. There were no significant differences in any of these variables for BFR‐leg SI and BFR‐leg LI (*P* = 0.920–0.998) nor for CTRL‐leg SI and CTRL‐leg LI (*P* = 0.408–1.0) throughout exercise (Figure 1). There was a leg × time interaction effect for QT_10:100_, indicating declines from pre‐ to post‐exercise for BFR‐leg SI (*P* < 0.001) and BFR‐leg LI (*P* = 0.004), but not in CTRL‐legs (Figure 1e). There were main effects of time for HRT and VA (both *P* < 0.001), indicating declines throughout exercise. Exercise did not lead to differences in VL and VM *M*_max_, but a slight decrease in RF *M*_max_ was observed over the course of exercise (main effect of time: *F*_(3,66)_ = 3.469, *P* = 0.024, η_p_^2^ = 0.126). A slight decrease for RF RMS_MVC_ was also observed (main effect of time: *F*_(4,113)_ = 3.122, *P* = 0.015, η_p_^2^ = 0.107), but not for VL or VM RMS_MVC_.

**TABLE 2:** Baseline neuromuscular characteristics by condition (*n *= 8).

	Short interval trial		Long interval trial		One‐way ANOVA between conditions	
Variable	CTRL‐leg	BFR‐leg	CTRL‐leg	BFR‐leg	*F*	*P*
MVC (N)	667 ± 195	645 ± 208	646 ± 168	667 ± 195	0.028	0.993
QT_single_ (N)	202 ± 74	206 ± 82	187 ± 51	205 ± 72	0.172	0.913
QT_10_ (N)	293 ± 108	294 ± 91	287 ± 80	291 ± 93	0.009	0.999
QT_100_ (N)	1.02 ± 0.12	1.05 ± 0.09	1.06 ± 0.13	1.04 ± 0.07	0.077	0.972
QT_10:100_ ratio	1.02 ± 0.12	1.05 ± 0.09	1.06 ± 0.13	1.04 ± 0.07	0.182	0.907
VA (%)	96 ± 2	95 ± 2	96 ± 2	96 ± 2	0.676	0.580
MRFD (N s^−1^)	6614 ± 3192	4242 ± 2415	6833 ± 2467	7262 ± 3246	2.041	0.150
HRT (s)	0.093 ± 0.013	0.087 ± 0.012	0.098 ± 0.012	0.089 ± 0.015	1.189	0.346
VL *M*_max_ (mV)	7.2 ± 2.5	7.4 ± 2.8	7.4 ± 2.8	7.3 ± 2.9	0.027	0.994
VM *M*_max_ (mV)	8.3 ± 2.1	8.8 ± 1.8	8.5 ± 2.2	8.4 ± 2.3	0.083	0.968
RF *M*_max_ (mV)	3.0 ± 1.1	3.0 ± 1.0	3.4 ± 1.3	3.5 ± 0.8	0.452	0.719
VL RMS_MVC_ (mV ms^–1^)	0.230 ± 0.086	0.238 ± 0.082	0.227 ± 0.092	0.297 ± 0.072	0.022	0.995
VM RMS_MVC_ (mV ms^–1^)	0.273 ± 0.068	0.282 ± 0.056	0.282 ± 0.073	0.288 ± 0.070	0.037	0.990
RF RMS_MVC_ (mV ms^–1^)	0.222 ± 0.044	0.229 ± 0.046	0.214 ± 0.039	0.216 ± 0.051	0.155	0.925

*Note*: Values are presented as group means ± SD and were analysed using a one‐way ANOVA to determine any difference in variables. Abbreviations: BFR, blood flow restriction, CTRL, control; HRT, half‐relaxation time; *M*_max_, M wave maximal amplitude; MRFD, maximum rate of force development; MVC, maximal voluntary contraction; QT_single_, QT_10_ and QT_100_, potentiated twitch peak force evoked by single, 10 Hz paired, and 100 Hz paired electrical stimulation, respectively; QT_10:100_, index of prolonged low‐frequency force depression; RF, rectus femoris; RMS_MVC_, root mean square of the surface electromyography signal during maximal voluntary contraction; VL, vastus lateralis; VM, vastus medialis.

### 3.3 Neuromuscular responses to post‐exercise recovery

**FIGURE 2:**
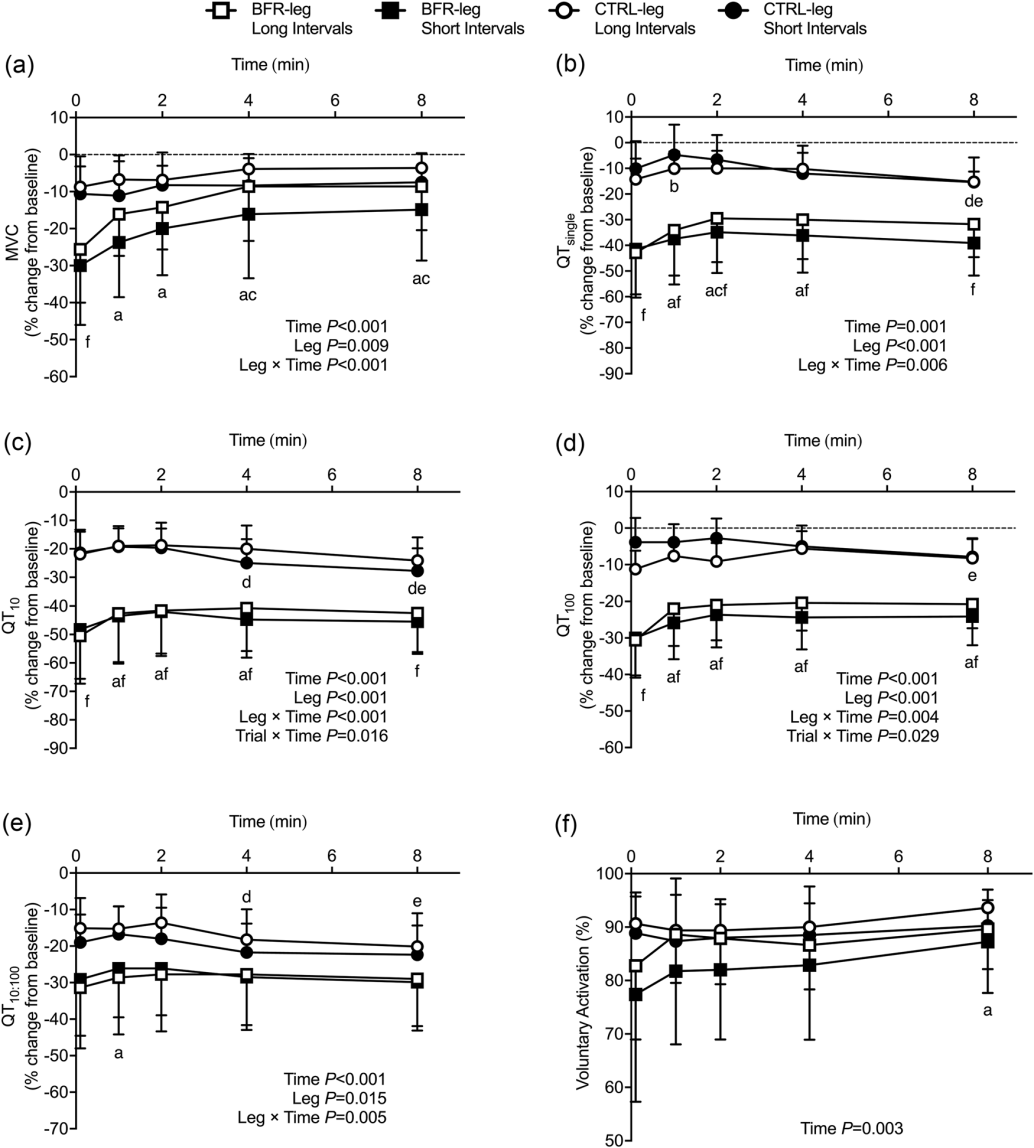
Effect of short‐interval and long‐interval trials, with (BFR‐leg) or without (CTRL‐leg) blood flow restriction throughout the post‐exercise recovery period, on maximal voluntary contraction (MVC, a), on potentiated twitch peak force evoked by single (QT_single_, b), 10 Hz (QT_10_, c) and 100 Hz (QT_100_, d) paired electrical stimulation, on prolonged low‐frequency force depression index (QT_10:100_, e) and on voluntary activation (f). Statistically significant main effects and interaction effects are indicated on the graphs. ^a^ *P* < 0.05 versus 10 s post‐exercise in BFR‐legs; ^b^ *P* < 0.05 versus 10 s post‐exercise in CTRL‐legs; ^c^ *P* < 0.05 versus 1 and/or 2 min post‐exercise in BFR‐legs; ^d^ *P* < 0.05 versus 1 and/or 2 min post‐exercise in CTRL‐legs; ^e^ *P* < 0.05 versus 4 min post‐exercise in CTRL‐leg; ^f^ *P* < 0.05 for BFR‐legs compared with CTRL‐legs.

MVC increased from post‐exercise to the end of the recovery period for BFR‐leg SI and BFR‐leg LI (both *P* < 0.001), but not in CTRL‐legs (Figure 2a). QT_single_ (*P* = 0.007, *d* = 1.8; *P* < 0.001, *d* = 2.08), QT_10_ (*P* = 0.003, *d* = 1.83; *P* = 0.004, *d* = 1.91), QT_100_ (*P* < 0.001, *d* = 2.07; *P* < 0.001, *d* = 2.74) and MRFD (*P* = 0.009, *d* = 1.95; *P* = 0.003, *d* = 1.46) were reduced to a greater extent throughout the post‐exercise recovery period in BFR‐leg SI compared with CTRL‐leg SI, and in BFR‐leg LI compared with CTRL‐leg LI. There were no differences in responses to post‐exercise recovery between SI and LI for the BFR‐leg (*P* = 0.859–1.0) or for CTRL‐leg (*P* = 0.693–0.989) in any of these variables. There were significant increases (i.e., recovery) compared with post‐exercise values in QT_single_, QT_10_ and QT_100_ during the recovery period for BFR‐leg SI and BFR‐leg LI, but not for CTRL‐legs (Figure 2b‐d). In general, most of the recovery in the BFR‐legs (between 3% and 10%) occurred within the first 1–2 min and levelled off thereafter until 8 min. These indices were only partially restored compared with baseline throughout the 8 min post‐exercise recovery period (all *P* < 0.05). The QT_10:100_ values remained less in the BFR‐legs compared with CTRL‐legs (*P* = 0.015, *d* = 0.92; Figure 2e) throughout the recovery period. There were significant main effects of time, indicating improvements during the post‐exercise recovery period, for HRT (*P* = 0.020) and VA (*P* = 0.005). The VA response significantly increased from post‐exercise to 8 min for BFR‐legs (*P* = 0.012, *d* = 0.62), but not for CTRL‐legs (Figure 2f). Interaction effects and main effects were not statistically significant for RMS_MVC_ and *M*_max_ in either the VL, VM or RF.

### 3.4 Near‐infrared spectroscopy variables

**FIGURE 3:**
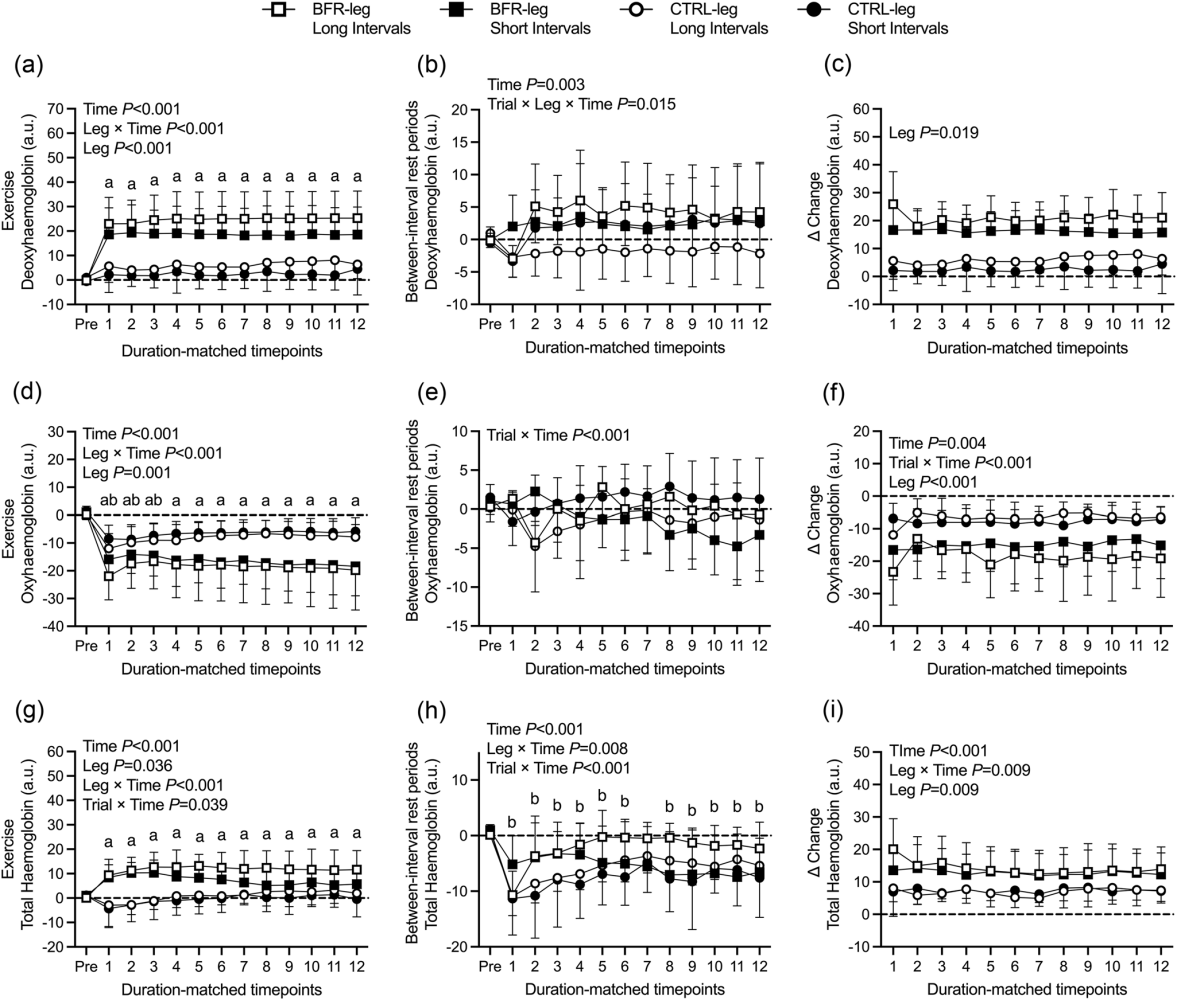
Effect of short‐interval and long‐interval trials, with (BFR‐leg) or without (CTRL‐leg) blood flow restriction, on deoxyhaemoglobin (a–c), oxyhaemoglobin (d–f) and total haemoglobin concentrations (g–i) during exercise (left column) and during between‐interval rest periods (central column). Data are also presented as Δ change from between‐interval rest periods to exercise (i.e., first exercise time point *minus* first between‐interval time point; right column). Statistically significant main effects and interaction effects are indicated on the graphs. ^a^ *P* < 0.05 versus baseline (Pre) in BFR‐legs; ^b^ *P* < 0.05 versus baseline (Pre) in CTRL‐legs.

Deoxyhaemoglobin and total haemoglobin concentrations during exercise (Figure 3, left column) were higher for BFR‐leg SI than CTRL‐leg SI (*P* = 0.004, *d* = 1.74; *P* = 0.042, *d* = 1.31), and for BFR‐leg LI than CTRL‐leg LI (*P* < 0.001, *d* = 2.14; *P* = 0.003, *d* = 1.81). No significant change between conditions in oxyhaemoglobin were found (*P* = 0.072–0.09; Figure 3, left column). During rest periods (Figure 3, middle column), there were no differences in the concentrations of deoxyhaemoglobin, oxyhaemoglobin or total haemoglobin for BFR‐leg SI and CTRL‐leg SI (*P* = 0.502–0.996) or for BFR‐leg LI and CTRL‐leg LI (*P* = 0.136–0.884). The Δ change from rest to exercise periods (Figure 3, right column) in deoxyhaemoglobin was more pronounced for BFR‐leg SI than CTRL‐leg SI (*P* < 0.001, *d* = 2.00) and for BFR‐leg LI than CTRL‐leg LI (*P* < 0.001, *d* = 2.23). It was also more pronounced in oxyhaemoglobin for BFR‐leg LI than CTRL‐leg LI (*P* = 0.014, *d* = 1.51), but not for BFR‐leg SI compared with CTRL‐leg SI (*P* = 0.211). There were no differences in the Δ change in total haemoglobin for BFR‐leg SI and CTRL‐leg SI (*P* = 0.313) or for BFR‐leg LI and CTRL‐leg LI (*P* = 0.138). There were no significant differences in deoxyhaemoglobin, oxyhaemoglobin or total haemoglobin for any of these variables between BFR‐leg SI and LI (*P* = 0.452–0.986) or between CTRL‐leg SI and LI (*P* = 0.660–0.999). The time spent over 90% of the maximal deoxyhaemoglobin was greater in BFR‐leg LI (386 ± 279 s vs. 129 ± 133 s; T_(7)_ = 2.49, *P* = 0.042, *d* = 1.18) than BFR‐leg SI.

### 3.5 Cardiovascular, respiratory, metabolic and perceptual variables

**FIGURE 4:**
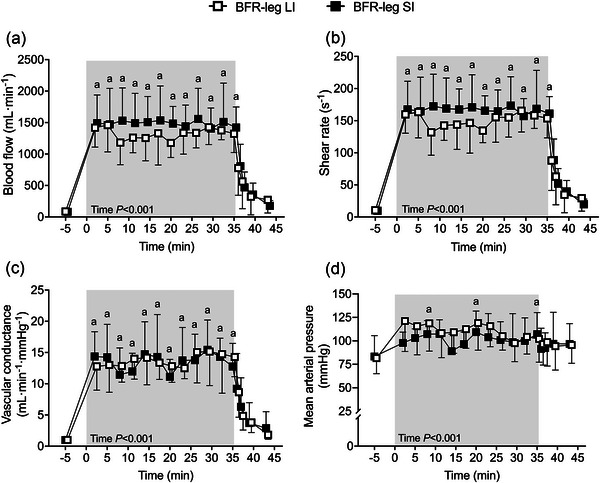
Blood flow (a), shear rate (b) and vascular conductance (c) from the leg exercising with blood flow restriction (BFR‐leg) and mean arterial pressure (d) upon cuff release. Data are shown at rest (i.e, ‐5 min), during (represented by shaded areas) and after short‐interval (SI) and long‐interval (LI) trials. Statistically significant main effects and interaction effects are indicated on the graphs. ^a^ *P* < 0.05 versus baseline in SI and LI.

Cardiovascular responses (see Figure 4) were quantified for seven of the eight participants due to difficulty obtaining measurements in one participant. Blood flow, shear rate, vascular conductance and MAP (main effects of time: *F* = 3.51–70.51, all *P* < 0.001, η_p_^2^ = 0.260–0.876) demonstrated an increase from baseline to exercise and decrease from exercise throughout the recovery period. No trial (*P* = 0.432–0.914) or trial × time interactions (*P* = 0.068–0.607) were evident in these measurements.

All cardiorespiratory variables (see Table 3) increased significantly over the course of exercise compared with baseline (*P* < 0.05) but remained unaffected by the nature of the cycling interval protocol (i.e., SI vs. LI; trial and interaction effects: *P* > 0.05). Blood lactate concentration was similar following LI and SI (3.8 ± 1.0 mmol ^–1^ vs. 3.3 ± 0.9 mmol ^–1^; *P* = 0.094).

**TABLE 3:** Metabolic and respiratory variables at baseline and during the exercise protocols (*n* = 8).

	Short intervals		Long intervals		Time effect	Trial effect	Interaction
Variable	Baseline	Exercise	Baseline	Exercise	*P* (η_p_^2^)	*P* (η_p_^2^)	*P* (η_p_^2^)
${\dot{V}}_{O_{2}}$ (L min^−1^)	0.32 ± 0.07	2.05 ± 0.48^*^	0.35 ± 0.06	1.94 ± 0.41^*^	<0.001 (0.951)	0.728 (0.009)	0.506 (0.032)
${\dot{V}}_{O_{2}}$ (mL kg^−1^ min^−1^)	4.6 ± 0.5	28.7 ± 3.0^*^	5.0 ± 0.8	27.4 ± 4^*^	<0.001 (0.983)	0.644 (0.016)	0.330 (0.068)
${\dot{V}}_{CO_{2}}$ (L min^−1^)	0.4 ± 0.06	1.98 ± 0.42^*^	0.4 ± 0.07	1.97 ± 0.4^*^	<0.001 (0.978)	0.883 (0.003)	0.800 (0.005)
*V̇*_E_ (L min^−1^)	12 ± 1	69 ± 17^*^	11 ± 2	66 ± 12^*^	<0.001 (0.950)	0.634 (0.017)	0.632 (0.017)
*V̇*_E_/${\dot{V}}_{CO_{2}}$	29.7 ± 2.8	35.0 ± 3.6^*^	28.6 ± 2.4	33.5 ± 2.5^*^	<0.001 (0.687)	0.267 (0.298)	0.803 (0.005)
RER	0.84 ± 0.05	1.0 ± 0.04^*^	0.82 ± 0.02	1.0 ± 0.04^*^	<0.001 (0.916)	0.596 (0.021)	0.599 (0.020)
*f*_B_ (breath min^−1^)	15.1 ± 3.1	45.1 ± 5.9^*^	14.0 ± 2.8	45.9 ± 8.5^*^	<0.001 (0.943)	0.931 (0.001)	0.643 (0.016)
*V*_T_ (L)	0.63 ± 0.12	1.82 ± 0.44^*^	0.67 ± 0.18	1.76 ± 0.46^*^	<0.001 (0.910)	0.930 (0.001)	0.617 (0.018)
*f*_B_/*V*_T_	16.9 ± 8.1	35.7 ± 14.9^*^	16.2 ± 6.0	38.8 ± 17.9^*^	<0.001(0.751)	0.833 (0.003)	0.562 (0.125)
HR (beats min^–1^)	75 ± 10	153 ± 9^*^	78 ± 9	155 ± 12^*^	<0.001 (0.991)	0.605 (0.020)	0.903 (0.001)

*Note*: Values are presented as means ± SD during pre‐exercise rest (baseline) and during cycling (over the course of exercise). Data were analysed using two‐way repeated measure ANOVA with trial (short intervals vs. long intervals) and time (rest vs. exercise) to determine the difference in variables between trial and time. ^*^Significantly different from baseline (*P* < 0.05). Abbreviations: *f*_B_/*V*_T_, breathing frequency/tidal volume ratio; HR, heart rate; RER, respiratory exchange ratio; ${\dot{V}}_{CO_{2}}$, carbon dioxide output; *V̇*_E_, minute ventilation; ${\dot{V}}_{E}$/${\dot{V}}_{CO_{2}}$, minute ventilation/carbon dioxide output ratio; ${\dot{V}}_{O_{2}}$, rate of oxygen consumption.

RPE and pain increased throughout exercise (i.e., main effect of time: both *P* < 0.001). On average, pain was ∼20% more (6.8 ± 1.5 a.u. vs. 5.6 ± 1.2 a.u.; *P* = 0.014, *d* = 0.88) for BFR‐leg LI compared with BFR‐leg SI.

## 4 DISCUSSION

The present study assessed acute changes in neuromuscular function, tissue oxygenation and cardiovascular responses over the course of short‐interval (SI) and long‐interval (LI) cycling exercise with BFR and without. The main findings in this experiment were that (1) exercise‐induced neuromuscular fatigue developed mainly following the initial repetitions in both limbs regardless of interval duration (Figure 1); (2) fatigue was further exacerbated throughout the course of exercise in the BFR‐leg in both SI and LI conditions, as demonstrated by greater reductions in MVC and potentiated evoked forces, while no additional changes were demonstrated in the CTRL‐leg (Figure 1); (3) BFR‐leg in both SI and LI resulted in more rapid recovery of neuromuscular fatigue despite remaining more pronounced compared to CTRL‐legs (Figure 2); and (4) no significant differences in the magnitude of the exercise‐induced hyperaemic response with BFR were found when considering the duration of multiple SI exercise compared with multiple LI exercise (Figure 4), whereas longer exposure to low levels of intramuscular oxygenation were observed during LI compared to SI exercise. The observed alterations in neuromuscular function, muscle oxygenation and blood flow that occur under specific testing conditions provide valuable insights into potential future applications of combining BFR with aerobic exercise.

### 4.1 Neuromuscular fatigue development during exercise

The current study utilized post‐exercise neuromuscular function assessments with a minimal time delay (i.e., exactly 10 s), which allowed us to accurately capture the time course and determine the aetiology of exercise‐induced neuromuscular fatigue development and recovery from BFR exercise. A significant decline in maximal voluntary force was found in both short‐duration and long‐duration intervals after the initial interval, which was more pronounced for the BFR‐leg than CTRL‐leg. A substantial portion of this exercise‐induced fatigue was of peripheral origin, as evidenced by a decrease in evoked twitch forces in the absence of voluntary activation deficits. The decrease in MVC/evoked forces after the first exercise intervals in the BFR‐legs accounted for approximately two‐thirds of the total reduction at exercise termination, showing that most of the exercise‐induced fatigue developed during the initial interval. This finding is consistent with data showing that the exercise‐induced decrease in the potentiated twitch was primarily observed during the initial phase of low‐intensity isotonic resistance exercise with BFR (Husmann et al., 2018), cycling time trial (de Almeida Azevedo et al., 2019) or repetitive concentric knee extension/flexion time trial (Froyd et al., 2013).

Following the initial reduction in MVC/evoked twitch forces after the initial intervals, the increase in muscle work was accompanied by the progressive development of neuromuscular fatigue in the BFR‐legs. This was evidenced by gradual declines in MVC, evoked twitch forces, VA, and RMS_MVC_ in the RF muscle. Meanwhile, the level of neuromuscular fatigue in the CTRL‐leg remained largely unchanged from the initial interval, suggesting that the rest periods between intervals allowed for sufficient recovery from the preceding exercise bout in the CTRL‐leg but not in the BFR‐leg. Moreover, for a given amount of work, the level of exercise‐induced neuromuscular fatigue was not different between SI and LI protocols, even though the time spent over 90% of the maximal deoxyhaemoglobin was greater in BFR‐leg LI. These findings indicate that during exercise with BFR, neuromuscular fatigue development is primarily linked to the amount of work during exercise, rather than the duration of exposure to lower levels of intramuscular oxygenation and/or the work–rest interval durations. Our results, which characterized neuromuscular fatigue development during repeated intervals of aerobic exercise, are consistent with previous findings obtained during four sets of low intensity resistance knee‐extension exercise (Husmann et al., 2018), demonstrating a progressive reduction in MVC and evoked twitch torque in the BFR condition.

By the end of exercise, the overall decrease in MVC/evoked forces reached 2.5–4 times greater in the BFR‐leg compared to the CTRL‐leg (Figure 1). Our findings extend previous results showing accentuated neuromuscular fatigue following dynamic leg extension resistance exercise (Husmann et al., 2018; Karabulut et al., 2010) and continuous cycling exercise (Kilgas et al., 2022) with BFR compared to without BFR. Although muscle metabolites were not measured herein, greater alterations in muscle contractile function in the BFR‐leg trials could be the result of reduced muscle O_2_ delivery (see Figure 3) and associated increase in intramuscular metabolic strain, such as increased inorganic phosphate (P_i_) (Suga et al., 2009; Sugaya et al., 2011). The accumulation of P_i_ can impair actin–myosin cross‐bridge cycling, ATP‐regulating Ca^2+^ uptake from the sarcoplasmic reticulum (Duke & Steele, 2000) and sarcoplasmic reticulum Ca^2+^ release, which collectively contribute to the impairment of excitation–contraction coupling (Allen et al., 2008). The decrease in twitch amplitude was not associated with changes in *M*_max_ recorded in the BFR‐leg, suggesting that reduced membrane excitability did not contribute to the further reduction in resting twitch amplitude in BFR‐legs compared to CTRL‐legs (Fuglevand et al., 1993). In addition to peripheral fatigue, central fatigue was also present throughout all conditions. Given the observation that more peripheral fatigue was induced by exercise in the BFR‐legs compared to CTRL‐legs, we interpret the similar decrease in voluntary activation throughout exercise as evidence that the further reduction in MVC in the BFR leg was the result of compromised muscle contractile function (i.e., peripheral fatigue).

### 4.2 Neuromuscular recovery kinetics

Short‐term (i.e., 10 s to 8 min) recovery from neuromuscular fatigue was significantly different between the BFR‐legs and CTRL‐legs, but not between SI and LI (Figure 2). This latter observation confirms that the metabolic and neuromuscular challenges in the BFR‐legs during exercise was similar between trials, despite different work–rest interval durations.

Peripheral fatigue in the BFR‐legs reached maximum levels 10 s after exercise termination. The alterations in the BFR‐legs in this study are consistent with the magnitude of exercise‐induced fatigue previously reported following short duration (6–10 min) cycling time trials in a population with similar anthropometric and physiological characteristics (Ducrocq & Blain, 2022). We interpret our findings as indirect evidence that the neuromuscular consequences of low intensity exercise with BFR mimic the perturbations observed during severe intensity cycling exercise. Herein, we found that the time course on post‐exercise recovery from neuromuscular fatigue showed a biphasic pattern, whereby a rapid but partial recovery was first observed mainly during the 10 s to 2 min period, followed by a tendency of voluntary and evoked forces to plateau without reaching baseline values after 8 min of recovery. Specifically, the overall ∼16% improvement in MVC during the post‐exercise recovery period may be explained by a ∼10% recovery in evoked twitch force during this time window. It is possible that the rapid restitution of peripheral fatigue indices might be explained by the increased reperfusion following BFR exercise (see Figure 4), which likely facilitated the removal of metabolites such as inorganic phosphate (Suga et al., 2012). Regarding central fatigue during the post‐exercise recovery period, we observed an enhancement in BFR‐leg VA, suggesting a gradual improvement, though not complete, in the central nervous system's capacity to activate muscles. These observations are aligned with previous studies showing rapid but incomplete short‐term recovery patterns in quadriceps MVC and evoked forces/torques following both resistance (Husmann et al., 2018) and aerobic (Kilgas et al., 2022) exercise with BFR.

With regards to the magnitude and pattern of neuromuscular recovery in the CTRL‐leg, minimal restitution of voluntary and stimulated forces were observed after exercise termination. Previous findings have demonstrated that the early phase of recovery from neuromuscular fatigue after exercise (i.e., 1–2 min post‐exercise) is mainly driven by the washout of metabolites associated with phosphocreatine hydrolysis and ATP depletion (i.e., P_i_ and ADP) (Baker et al., 1993; Bogdanis et al., 1995; Miller et al., 1987). We interpret these findings as evidence that the accumulation of these metabolites during exercise in the BFR‐leg was larger than in the CTRL‐leg and, therefore, the need for a metabolite washout period after exercise was much lower in the CTRL‐leg compared to the BFR‐leg. This interpretation is supported by previous findings showing that low‐intensity BFR training elicits significantly greater metabolic stress in skeletal muscle than the same exercise without BFR (Suga et al., 2009). Moreover, compared with CTRL‐legs, peripheral muscle fatigue in the BFR‐legs remained exacerbated throughout recovery, at least up to 8 min post‐exercise. Our findings showing that QT_10:100_ remained less throughout recovery after exercise with BFR compared to CTRL suggest that the BFR‐legs were influenced more by mechanisms associated with prolonged low‐frequency force depression. This might be explained, at least in part, by impaired excitation–contraction coupling and muscle Ca^2+^ handling/sensitivity from the sarcoplasmic reticulum in response to reactive oxygen species production associated with repeated exposures to low intramuscular O_2_ partial pressure during BFR (Bruton et al., 2008; Cheng et al., 2015; Watanabe & Wada, 2016).

### 4.3 Cardiovascular and muscle oxygenation responses to BFR exercise

One purpose of the present study was to determine whether manipulating the number of episodes of BFR would affect the magnitude of muscle O_2_ availability and shear stress during exercise and/or recovery, two potent mediators of the BFR training‐induced muscle and vascular adaptations (Christiansen, 2019; Hudlicka & Brown, 2009; Tinken et al., 2010). The reduction in muscle blood perfusion during exercise with BFR (Mouser et al., 2017) led to a marked reduction in muscle oxygenation and increase in muscle O_2_ extraction in the vastus lateralis. These changes were indicated by increases in deoxyhaemoglobin (Figure 3a) and reductions in oxyhaemoglobin (Figure 3d) throughout exercise. The observation that tissue oxygenation decreased between 22% and 26% in the present study falls within previously reported ranges of 20%–45% in oxyhaemoglobin and deoxyhaemoglobin during low‐load resistance exercise (15%–20% one repetition maximum) with BFR, performed to task failure with full (Kacin & Strazar, 2011) or partial (Kolind et al., 2023) arterial occlusion. During the brief recovery periods between intervals, the increase in blood flow that accompanied the rise in vascular conductance upon cuff release (Figure 3) contributed to restored tissue oxygenation levels and promoted vascular shear stress, as evidence by the ∼10 times increase in shear rate upon cuff release compared to baseline with free flow. The hyperaemic response to cuff release was similar throughout trials, indicating that 1 min  of BFR during low intensity cycling exercise was sufficient to maximize shear stress. Shear stress on the arterial walls combined with metabolic perturbations and reductions in tissue oxygen tension during BFR represent potent stimuli to the remodelling of conduit and resistance arteries, the improvement in endothelial function, muscle capillary growth and/or mitochondrial biogenesis (Green et al., 2017; Krustrup et al., 2009; Prior et al., 2004; Suga et al., 2009). We also observed that the marked increase in femoral vascular conductance associated with increased blood flow did not lead to a decrease in MAP, a crucial factor in preserving brain perfusion. This finding suggests that either cardiac output was adequate to prevent a pressure drop upon cuff release or that vascular conductance was constrained from further increasing by the exercise pressor reflex (Thurston et al., 2023), which is markedly heightened during exercise with BFR (Hori et al., 2021).

### 4.4 Implications

The results of this investigation showed that cycling with BFR can induce neuromuscular impairments comparable to that observed during severe intensity cycling exercise without BFR. These impairments, indicative of severe intramuscular perturbations, occurred in the presence of reduced muscle oxygenation during BFR and accentuated shear stress at cuff release. Such stimuli are recognized for their central role in exercise‐induced improvements in vascular and muscle functions (Ferguson et al., 2021; Pignanelli et al., 2021). We also observed that within 2:1 work–rest ratio intervals and for a given amount of work, exercise‐induced neuromuscular fatigue development, the magnitude of shear stress and muscle oxygenation patterns were either not significantly influenced or were only slightly affected by different work–rest durations. In contrast, our results indicated that pain, a sensation commonly associated with BFR exercise (Kilgas et al., 2019; McClean et al., 2023), was greater with longer compared to shorter intervals, suggesting a reduced tolerance to extended BFR durations. Based on these findings, we propose that administering short intervals with BFR may allow for a greater amount of total work to be performed before reaching failure or eliciting the same level of fatigue but with the advantage of integrating more alternate periods of metabolic perturbations and shear stress within similar training durations. Consequently, future research should focus on assessing the chronic effects of short duration interval training with BFR on improvements in cardiovascular and muscle function as well as exercise tolerance and BFR acceptance.

### 4.5 Limitations

There are several limitations to be addressed in the current study. Despite instructions, familiarization and real‐time visual feedback, distribution of power output during exercise was 55% versus 45% in the CTRL‐leg compared to the BFR‐leg in both trials. Our fatigue results might thus overestimate and underestimate fatigue in the CTRL‐leg and BFR‐leg, respectively. Moreover, we cannot exclude the possibility that the neuromuscular perturbations to the BFR‐leg could negatively affect the force‐generating capacity of the contralateral CTRL‐leg via a quadriceps neuromuscular fatigue cross‐over effect (Halperin et al., 2015). However, several data exist suggesting that cross‐over fatigue (measured via reduced contractile function or increased central fatigue) might be limited in the lower limb muscles (Aboodarda et al., 2019, 2020; Amann et al., 2013). It is also noteworthy that while occlusion pressure was measured in triplicate to ensure accuracy, it is possible that some between‐day variability influenced relative arterial occlusion pressures (Ingram et al., 2017). However, it has been previously shown that there is limited (i.e., <4.5%) difference in between days AOP measurements (Ingram et al., 2017).

### 4.6 Conclusions

In this study, in which assessments of neuromuscular function were performed on both limbs simultaneously, we were able to differentiate the acute exercise‐induced neuromuscular responses between exercise with BFR and exercise without. We provide evidence that cycling with BFR elicits a greater magnitude of quadriceps neuromuscular fatigue than cycling without BFR, independent of the exercise/recovery duration. Specifically, higher levels of peripheral fatigue and faster restitution during its early recovery phase were found with BFR exercise compared to exercise without BFR. These findings are consistent with a greater buildup of metabolites during BFR exercise than in exercise without BFR. Moreover, BFR was associated with a significant reduction in muscle oxygenation and increase in sheer stress following cuff release, with the work–rest duration having little, if any, influence on these responses. Together, our results provide insights into the applications of cycling exercise with BFR, which may aid researchers and practitioners in developing protocols for both healthy and clinical populations.

## AUTHOR CONTRIBUTIONS

Colin Lavigne, Valentin Mons, and Grégory M. Blain contributed to the conception and design of the research. Colin Lavigne and Maxime Grange performed the experiments. Colin Lavigne, Valentin Mons, and Maxime Grange analysed the data. Colin Lavigne and Grégory M. Blain interpreted the results of the experiments. Colin Lavigne drafted the manuscript and prepared the figures. Colin Lavigne and Grégory M. Blain edited and revised the manuscript. All authors have read and approved the final version of this manuscript and agree to be accountable for all aspects of the work in ensuring that questions related to the accuracy or integrity of any part of the work are appropriately investigated and resolved. All persons designated as authors qualify for authorship, and all those who qualify for authorship are listed.

## CONFLICT OF INTEREST

None declared.

## FUNDING INFORMATION

None.

## Data Availability

Data are available upon reasonable request.
